# Stakeholders’ views and experiences on implementing new diagnostics in primary care to support management of community-acquired acute respiratory tract infections: a qualitative study

**DOI:** 10.3389/fpubh.2023.1216940

**Published:** 2023-07-31

**Authors:** Melanie Eugenie Hoste, Elien Colman, Marta Wanat, Gail Hayward, Jean-Louis Tissier, Maarten Postma, Herman Goossens, Sibyl Anthierens, Sarah Tonkin-Crine, on behalf of the VALUE-Dx study team

**Affiliations:** ^1^Department of Family Medicine and Population Health, University of Antwerp, Antwerp, Belgium; ^2^Laboratory of Medical Microbiology, Vaccine and Infectious Disease Institute, University of Antwerp, Antwerp, Belgium; ^3^Nuffield Department of Primary Care Health Sciences, University of Oxford, Oxford, United Kingdom; ^4^bioMérieux, Marcy l’Etoile, France; ^5^Unit of Global Health, Department of Health Sciences, University Medical Center Groningen, University of Groningen, Groningen, Netherlands

**Keywords:** point-of-care testing, respiratory tract infections, qualitative, primary care, antibiotic prescribing, stakeholder, Europe

## Abstract

**Background:**

The majority of antibiotics are prescribed in primary care for respiratory tract infections. Point-of-care tests (POCTs) for the management of community-acquired acute respiratory tract infections (CA-ARTI) have been developed to help optimize antibiotic prescribing. While some countries in Europe have adopted these tests in primary care settings, most have not. Stakeholders, such as policy-makers, regulators, the diagnostic industry, and scientific associations, have roles in the implementation of new diagnostics in primary care. The aim of this study is to explore these stakeholders’ views and experiences, and identify areas of unmet need relating to POCT implementation.

**Methods:**

Stakeholders were recruited using purposive sampling and snowballing. Between March 2021 and May 2022, semi-structured interviews were conducted online with stakeholders in Belgium, the UK and from European Union (EU) -level organizations. Interviews were audio recorded and transcribed verbatim. Transcripts were analysed inductively and deductively using thematic analysis.

**Results:**

Twenty-six stakeholders participated: eleven from EU-level organizations, seven from Belgium, and eight from the UK. Five themes were identified. Stakeholders felt a balance of top-down and bottom-up approaches were an optimal strategy to the implementation of POCTs. Stakeholders stressed the need to engage with clinicians to act as champions for tests to help raise awareness and generate new evidence on how tests are used. While acknowledging the potential of POCTs for improving patient outcomes and impacting antibiotic prescribing behavior, some raised concerns on how tests would be used in practice and wished to see national data on effectiveness. COVID-19 catalyzed the use of tests, but stakeholders were pessimistic that processes for approving diagnostics during the pandemic would be replicated in the future.

**Conclusion:**

Stakeholders provided recommendations for research and practice. Robust reimbursement policies could alleviate financial burden from clinicians and patients, encouraging practices to adopt POCTs. Industry is likely to benefit from engaging as early on as possible with other stakeholders. Due to uncertainty among stakeholders on the impact of POCTs on antibiotic prescribing, further evidence is needed to understand how practices adopt POCTs and the implications for stewardship. Monitoring how POCTs are used can inform future guidelines on successful diagnostic implementation.

## 1. Introduction

The majority of antibiotics in Europe are prescribed in primary care settings for community-acquired acute respiratory tract infections (CA-ARTI) which are often self-limiting and viral in etiology (1, 2). While antibiotic surveillance and stewardship programs exist in Europe, significant variation in antibiotic prescribing between, and within, countries persists (3). Despite existing literature supporting the use of point-of-care tests (POCTs) as an intervention to safely reduce antibiotic prescribing for CA-ARTI in primary care, only a few countries in Europe have introduced them in primary care (4, 5).

A POCT in primary care is defined as a test performed during a consultation by a healthcare professional with results made available at the time of patient presentation to support clinical decision-making (6). Some European countries such as Norway, Sweden, the Netherlands, Germany, the Czech Republic, Estonia, and Switzerland have included point-of-care C-reactive protein in their national guidelines on the management of CA-ARTI (7). Other POCTs such as rapid antigen detection tests for group A streptococcus are included in national guidelines for France, Germany, Italy, Spain, and Sweden (8). Despite agencies such as the National Institute of Health and Care Excellence and the European Respiratory Society recommending POCTs in their guidelines, these diagnostics are seldom widely adopted across European primary care settings (9).

Due to the complexity behind implementing diagnostics, the contexts where POCTs are implemented need to be understood. Previous qualitative research focussing on the views of clinicians using POCTS for CA-ARTI have stressed the importance of reimbursement policies, inclusion of POCTs in guidelines, diagnostics that fit into existing workflows, and the support from management for primary care to adopt POCTs (10–12). Qualitative studies understanding the perspectives of stakeholders who are instrumental to the implementation process, therefore, may provide insights into some of the factors that influence uptake of POCTs in primary care settings. To date, evidence on barriers and facilitators to implementing CA-ARTI-specific POCTs from the perspectives of stakeholders in Europe have been limited to studies in the UK (7, 13, 14). UK studies on stakeholders, that held similar roles to those recruited in this study, were conducted prior to the pandemic or focussed on pediatric ambulatory care (7, 13, 14). These studies illustrated that technology design, stakeholder engagement, and sufficient evidence on utility aid adoption. In addition, the COVID-19 pandemic has triggered public recognition and investment in POCTs (15, 16) which may influence adoption of other tests. This study examines European stakeholders’ views and experiences of implementing novel POCTs in primary care, and their views on the impact of the COVID-19 pandemic in this area. Our objective was to identify how the adoption of diagnostics can be supported at both organization and system levels. We sought to provide recommendations that would assist policy-makers and the diagnostic industry in effectively implementing POCTs for CA-ARTI.

## 2. Methods

### 2.1. Study design and participant recruitment

This qualitative study was designed as part of the *Value of diagnostics to combat antimicrobial resistance by optimizing antibiotic use* (VALUE-Dx) program (17).

We wanted to interview people in roles relevant to the introduction of new diagnostics in primary care settings and who are familiar with POCTs for CA-ARTI including policy-makers, guideline developers, the diagnostic industry, reimbursement agencies, regulators, and clinician and pharmacy organizations. Authors (EC, MEH, ST-C, SA) identified potential stakeholders using both information available in the public domain and using the networks of members of the wider VALUE-Dx consortium. We chose to focus on stakeholders from European Union (EU) -level organizations, and in Belgium and the UK where the research team had existing established networks. Throughout this paper, we will refer to participants who work at an EU-level as EU participants.

EU stakeholders were recruited first, followed by stakeholders in Belgium and the UK. Initially, we planned to recruit stakeholders from the EU and from four European countries (Belgium, the UK, the Netherlands, and Sweden). We selected Belgium and the UK as countries with limited POCT implementation in primary care, and Sweden and the Netherlands as countries with established POCT implementation. However, we experienced difficulties contacting potential participants in Sweden and the Netherlands as a result of individuals being unavailable due to the COVID-19 pandemic. We confined our recruitment to the EU, Belgium, and the UK where the team had stronger existing networks and could approach more potential participants. In addition, we used snowballing sampling by asking interviewed participants if they could recommend a potential participant whose role would be relevant to this study.

### 2.2. Interviews

Potential participants were invited by e-mail with a participant information sheet. Interviews were conducted online on Microsoft Teams by author MEH, a PhD student with experience in qualitative methods, following a semi-structured topic guide (Supplementary material), with participants giving informed verbal consent at the start of interview. The topic guide was developed to ensure that key questions on implementation were asked to all participants, with the flexibility for follow-up questions as needed (18–20). VALUE-Dx researchers outside of the core research team reviewed the topic guide to ensure relevancy. Questions asked about the facilitators and barriers to, and any experience of, the adoption of diagnostics and current progress in implementation of diagnostics in primary care settings. In addition, specific questions were asked about the COVID-19 pandemic and the potential impact of the pandemic on future implementation of new diagnostics. All participants involved in this study were familiar with POCTs specifically for CA-ARTI due to their involvement with diagnostics and/or their expertise in antimicrobial stewardship. Participants from Belgium and the UK provided their perspectives on POCTs for CA-ARTI despite limited experience of implementing these types of tests and drew on previous experiences of implementing novel diagnostics. Interviews were conducted in English, audio-recorded, and transcribed verbatim.

### 2.3. Data analysis

Transcripts were pseudonymised and NVivo 12 was used to support analysis. An exploratory approach was used in the analysis to avoid imposing a pre-existing framework on the data. As the interviews with EU participants took place first, the core team read the interviews and immersed themselves in the data. Thematic analysis was used to analyse the transcripts (18). They were read line-by-line by author MEH and detailed codes were first created inductively for all EU transcripts and grouped to create categories and sub-categories. These categories and sub-categories were then discussed within the core team (MEH, SA, ST-C) and amended accordingly creating an initial data-driven framework based on the EU transcripts. Interviews of Belgian participants then took place and were coded deductively into the initial framework. Interviews with UK participants, and the subsequent analysis, started when interviews with Belgian participants concluded. Following the same analytical process, UK transcripts were coded deductively into the revised sub-categories and categories. Throughout our analysis, we also considered participants’ role and country when exploring similarities and differences between participants’ views.

Monthly core team discussions were held throughout the data collection and analysis phases to deliberate on the framework and to follow an iterative approach. These discussions served to refine and align the framework with the data from each country. This type of research triangulation brought together different perspectives allowing alternative interpretations of the framework to be considered (21). We referred to the quality criteria for qualitative criteria to ensure rigor and trustworthiness of our data (21, 22). A reflexive approach was adopted as ST-C and SA had prior experiences with qualitative studies on POCTs and were mindful of this during the analysis process. Themes and sub-themes were developed only after all transcripts had been analysed. Moreover, discussions were held with the wider research team of VALUE-Dx to discuss alternative interpretations of the framework.

## 3. Results

We conducted interviews with 26 participants between March 2021 and May 2022: eleven from EU organizations; seven from Belgium; and eight from the UK. The interviews lasted between 24 min and 70 min (mean 51 min). Table 1 presents both the number of participants interviewed and examples of job roles held by the participants at the time of the study.

**Table 1:** Information on participants.

	EU	Belgium	UK
Policy-maker (e.g., Head of directorate for a reimbursement body; Coordinator of an antibiotic policy committee; Member of a reimbursement agency)	2	4	5
Scientific association (e.g., Lead for diagnostics in a government funding body; Member of a pharmacy association; Medical directory of a general practice association)	5	3	2
Diagnostic industry (e.g., Chief scientific officer; Market access director)	4	0	1
Total number of participants	11	7	8

Five themes were identified, describing common experiences across all stakeholders.

### 3.1. Theme 1: policy-level influences to support implementation

All participants expressed the importance of having sufficient financial resources to successfully implement POCTs in primary care settings. They flagged that competition with other innovations and variations in funding across regions and countries, depending on government priorities, meant that funding for POCTs was inconsistent and variable. Participants from Belgium and the UK reported a lack of reimbursement strategies for POCTs in their countries, which they perceived as a barrier to the uptake of these tests by clinicians. Belgian participants suggested that a lump sum fee for diagnostics would be a possible solution for practices that would include the cost of performing a test.

*“What we are proposing for this kind of testing is that you're paying a kind of lump sum. So maybe it's €26 or €27 … So [the patient] pay[s] a certain amount, but everything is included … If you need a fee for every test, in the end, you will have to pay a big amount, but for the same result.” P15, Policy-maker, Belgium*

In the UK, participants noted that there was no established funding framework for integrating POCTs in the current primary care system. In addition, some UK participants reported that antimicrobial resistance was not seen to impact primary care directly and subsequently felt that clinicians would not want to accept the costs of implementing POCTs. They believed that bulk purchasing of POCTs and procurement on a national level, with delivery to practices, could alleviate some of these challenges.

*“General practitioners are independent contractors and if they had to purchase the tests and source them, supply them … I think that that's additional operational [work] and inconvenience for them whereas, if the health service at a national level purchased the tests on their behalf and delivered them to GP practices, they might be more inclined to use them but having to order them themselves would be another barrier.” P24, Policy-maker, UK*

EU participants wished to see a harmonization of health technology assessments (HTAs) across Europe either through a European body or a standard evaluation for diagnostics. They further elaborated that HTAs are not consistent across countries, leading to lengthy and time-consuming regulatory processes that technology developers must navigate. Some EU and UK participants reported that current regulations are likely obsolete and need to be updated to reflect the changing diagnostic market. They pointed to Europe’s CE-marking (a mark indicating that the product meets EU safety, health and environmental protection requirements) for *in vitro* diagnostics, for instance, as an insufficient standard for assessing diagnostics.

*“I think the real barrier for us is, as part of the notifiable body – so to get it to CE mark or CA-marking in the future, what needs to happen is that there needs to be a technical evaluation at that stage before. Because they get a CE-mark in this country on self-declaration. That is not good enough.” P26, Policy-maker, UK*

### 3.2. Theme 2: multi-level system approach to implementing POCTs

Participants from Belgium and the UK reported that a strategy that combines both a top-down and bottom-up approach to implementing POCTs in primary care settings might be best. They felt a top-down approach would be helpful for interventions that require resources to implement and possibly the revision of national guidelines to include POCTs to support adoption. In addition, EU participants stated that engaging with policy-makers at the European Commission to place diagnostics on Member States’ agendas can be a facilitator, prompting countries to reimburse POCTs and invest resources into supporting primary care in their implementation.

Belgian participants further believed that politics may have an active role in the implementation of POCTs. They illustrated that their country is divided into communities, which have authority over healthcare including prevention measures. If POCTs are classified as a preventive measure, it falls into the jurisdiction of the communities to make decisions.

*“[In Belgium] You have a federal organisation and federal healthcare, but you also have the communities … the French-speaking parts, Dutch-speaking parts, and the federated states, that have responsibilities in terms of healthcare. Also, they have the responsibility and the accountability for everything which is prevention. So if you consider diagnostics as a part of prevention than a part of therapy, especially if we're talking about screening … this might become a political discussion over who is responsible for it and who should pay for what. Who should decide: 'What types do we choose? Who pays the bill?” P16, Policy-maker, Belgium*

However, most participants highlighted that the drive to use POCTs in primary care needs to come from clinicians, as the main stakeholders, for successful implementation. Belgian and UK participants believed that clinicians have considerable influence, as early adopters, to motivate others to implement POCTs as they would be generating the evidence to show how POCTs could fit within clinical workflows.

*“I think they [clinicians] will also help drive the use of these tests as well because as the earlier adopters and innovators, they will help produce some of the evidence that will then be used to support policy at a national level and then filter out to wider adoption.” P25, Policy-maker, UK*

EU, Belgian, and UK participants believed that there is still a lack of awareness among clinicians on the existence of POCTs and how they can optimize prescribing. They recommended educating clinicians through seminars and training to demonstrate the effectiveness of POCTs.

On the other hand, participants maintained that having relevant stakeholders agree on how POCTs should be adopted is crucial but felt this was a challenge for technology developers as it can be time-consuming and costly to identify stakeholders. UK participants believed that a certain amount of lobbying was needed to engage with stakeholders for successful implementation which relied on trust and being connected to those who can help.

*“You have to do a lot of lobbying around entering NHS. But it's a very, very difficult process and usually how it works is, you know someone in the central commissioning group and they trust you and eventually they might agree to run a pilot, you gather some evidence from the NHS pilot and you can then implement it broader. And so this process is not very efficient because it all relies on knowing people and being connected to the right people.” P22, Industry, UK*

Most participants highlighted the importance of convincing arguments and narratives that demonstrate the value of POCTs to facilitate implementation. They stressed that evidence demonstrating advantages from a public health perspective, including improving day-to-day practice and clinical effectiveness, would strengthen arguments to support POCT implementation.

### 3.3. Theme 3: extent of POCTs contributing to optimal patient care in primary care

Most participants raised aspects of POCT technology such as accuracy and clinical effectiveness as crucial features to ensure they are adopted. However, some EU participants, particularly those from professional organizations and some from industry, believed that POCT technology may still need further improvements before tests can be widely adopted. Participants were concerned with the accuracy of test results and believed that faster diagnostics need to be developed.

*“I do believe that the companies still have a way to go, with the exception of COVID-19 where we’re seeing a lot of momentum, to actually develop more so easier to use, faster and more precise diagnostic tools so that the community can use.” P6, Industry, EU*

Moreover, there were mixed opinions across participants on the benefits of POCTs. Some participants explained that POCTs could potentially be beneficial to patients in primary care by optimizing antibiotic prescribing behavior and consequently, influencing clinician-patient relationships. They suggested that results from a test could be used by clinicians as a communication tool with patients to support their decision-making process. Others, on the other hand, believed that more real-world evidence is needed to demonstrate that POCTs are likely to optimize antibiotic prescribing. Belgian and UK participants also stressed the importance of country-specific data which they argued was currently lacking. They pointed to differences between different healthcare systems and stressed that existing studies, on cost-effectiveness, for example, may not be relevant for their own countries.

*“[With the] C-reactive protein test it’s not so clear if it's a good thing, or if it's a not a good thing to implement … We need scientific evidence.” P18, Scientific association, Belgium*

*“The NICE committees would often worry if evidence was just from the US, or evidence from China or Russia. There can be major differences in how the health system works … those costs and the way the system operates in the US isn't necessarily reflective of UK practice.” P19, Policy-maker, UK*

However, participants also reported that demonstrating cost-effectiveness of POCTs can be challenging especially when factoring in savings in healthcare costs in relation to antimicrobial resistance. They feared that other less costly strategies to reduce antibiotic prescribing may have not been extensively adopted in primary care and could be first introduced.

Interestingly, some Belgian and UK participants were skeptical about how POCTs would be used in real-life settings and were concerned about both the overuse and underuse of POCTs. Some argued that tests would not be used in practice if they are too expensive and cited research where POCTs were left unused in primary care. If POCTs were incentivized, some UK participants felt that this could lead to overuse thus, further guidance would be needed to instruct clinicians on when to use them.

*“We would want to incentivize GPs to use the test. But then we'd run the risk of over testing … But the question remains open as to what is the right proportion of patients that should have a diagnostic test and there's no clear answer to that question.” P24, Policy-maker, UK*

EU and UK participants suggested that implementing POCTs may be challenging in some practices as some clinicians may be more inclined to prescribe antibiotics. Some UK participants stated that POCTs may not change prescribing behavior as a test may not be able to fully rule out a bacterial infection. Others stated that making a conscious decision to test first, before indicating a prescribing decision, is challenging. In addition, they believed that patients may even insist on antibiotics regardless of test results.

*“I mean it is quite a big change to actually have do a test rather than prescribing some type of antibiotic out of habit. It’s breaking that habit.” P6, Industry, EU*

### 3.4. Theme 4: implementing POCTs will impact the current organization of primary care in Europe

Most participants generally agreed that implementing POCTs in primary care involves re-organizing the way services are run with barriers such as stretched workforces and limited consultation times.

*“I have the problem to have the time to use it because in my daily job [general practitioner] I have enough things to do. Then I need not only these tools in a point of care, but also that they are easy to use.” P8, Scientific association, EU-level*

*“If they've got a new patient in the door every ten minutes and, you know, there are some logistics, that they need a nurse to deliver the point-of-care test, and then that nurse then has to feed back the result to the GP to say: 'Oh, it's 150. Do you want to give an antibiotic?' You know. It kind of interferes potentially with the flow.” P19, Policy-maker, UK*

If POCTs are to be implemented in primary care, EU participants, including those from industry and professional organizations, argued that laboratories need to be involved. They felt that the existence of reference labs is important to ensure the quality of POCTs and to oversee data coming from the community. Interestingly, Belgian participants explained that because of the way laboratories are organized in Belgium, some laboratories would suffer a loss of income if POCTs were widely adopted in primary care.

*“In Belgium, you have a lot of labs. You have private labs and labs that are linked to the hospital, you have the clinical biologist, and the clinical biologists don't want GPs to do this kind of test because for them, it's a loss of income. If the GPs are using it, then for them, it's a loss of income. So there will be a lot of struggle, I think, a lot of discussions.” P16, Policy-maker, Belgium*

*“I think the [laboratories] would be against [the proposal of implementing POCTs] as the laboratories will lose money … If we [general practices] do point-of-care testing that means we will send less to the labs.” P18, Scientific association, Belgium*

### 3.5. Theme 5: perceived influence of COVID-19 on the future of POCT implementation

EU, Belgian, and UK participants reported that the pandemic provided a further understanding on how to implement diagnostics, including using pharmacies for testing to relieve pressure on primary care. While some expressed hope that pharmacies would implement POCTs in the future, Belgian participants recalled previous attempts where primary care was resistant as they wanted clarity in defining roles and concerns arose about costs of adoption.

*“We have experienced some pushback from physicians, both from physician organisations as well as the federal healthcare service and from the physicians. It seemed to be related to clear role definitions of who is doing what in terms of care, whereas from the federal healthcare service, they seem to be concerned also, with the cost of introducing those type of first-line [general practice] or zero-line settings [pharmacies] as an additional channel.” P12, Scientific association, Belgium*

Belgian and UK participants highlighted different lessons that were learnt as a result of the pandemic. In Belgium, for example, some participants reported learning about how evidence could be translated into practice in other countries. UK participants highlighted, on the other hand, that strengthening communications between stakeholders and sharing knowledge were seen as being essential for successful implementation of diagnostics. Furthermore, UK participants felt that the public was now more confident in using rapid antigen tests and familiar with certain terminology related to CA-ARTI. They also expressed that testing may no longer fall under the “gate-keeper” role of clinicians.

*“I think you know people are more familiar now with using lateral flow tests in the home and they're more familiar with certain terminology as well.” P24, Policy-maker, UK*

However, participants in Belgium and the UK voiced some doubts on the extent of the pandemic’s impact on implementing future diagnostics. They raised the concern that the pandemic was not a reflection of “normal” times as governments were under immense pressure to expand testing capacities as quickly as possible, bypassing some of the regulatory processes that would have usually been followed.

## 4. Discussion

### 4.1. Summary of main findings

This study presents the views and experiences of stakeholders involved in the implementation of diagnostics on an EU-level, and in Belgium and the UK. The interviewed stakeholders from Belgium and the UK provided their perspectives on POCT implementation in primary care, despite the limited experience of adopting POCTs for CA-ARTI at the time of the study. Our participants from different stakeholder groups agreed with each other on the factors that influence the implementation of POCTs for CA-ARTI in primary care.

Most participants agreed that top-down influences such as dedicated funding for diagnostics alongside policy changes would be needed to facilitate the adoption of POCTs in primary care. However, there was recognition that top-level changes alone may not be sufficient and that a bottom-up approach was needed in other areas in order to successfully implement. Participants noted that that the drive for POCT implementation should come from clinicians. This may require POCT “champions” engaging with clinicians to gather greater evidence showing patient benefits from using diagnostics.

Conversely, participants expressed doubts to the extent to which POCTs can improve primary care practice. Belgian and UK participants particularly wanted to see national evidence demonstrating the value of POCTs and were concerned over potential over-or under-use of POCTs which subsequently, may not change the prescribing behavior of clinicians in the long run. Some participants stated that the availability of POCTs alone may not be sufficient to ensure their appropriate use, suggesting that bottom-up approaches may be necessary to influence clinicians’ prescribing behavior.

In addition, participants acknowledged that adjustments may be needed in the ways primary care services are run in order to implement POCTs, which may be a logistical challenge. They believed that barriers such as restricted workforce and consultation times may need to be taken into account. In particular, Belgian participants raised concerns about the influence of introducing POCTs in primary care on the role played by laboratories, potentially resulting in a financial loss for them. Consequently, additional considerations may be necessary to determine the appropriate placement of laboratories within the care pathway.

Finally, participants had mixed opinions on whether the COVID-19 pandemic would have a significant impact on the implementation of future diagnostics. Participants in Belgium and the UK pointed out that processes and strategies to implement POCTs for COVID-19 could not be replicated for future diagnostics except under exceptional circumstances. However, some agreed that lessons can still be learnt such as opening up new settings for testing, building on relationships developed during the pandemic, and the public’s familiarity with testing.

### 4.2. Strengths and limitations

Using qualitative methods helped to unearth the complexity behind implementing POCTs from different stakeholders’ perspectives. Investigation from an EU-level stance provided a macro-level perspective of some of the challenges that technology developers have to face when navigating the European regulatory landscape; while national stakeholders provided important context. Although the interviews gathered rich data, recruiting other stakeholders such as patient groups would have added to the scope of the study by understanding if and how patients may have an influence on implementation. Interviewing stakeholders from European countries where the use of POCTs in primary care practices is routine could have offered a contrasting example to further understand how challenges were overcome and what facilitators supported POCT adoption in their contexts. We did attempt to recruit participants in other European countries where POCTs have been widely adopted, however due to the timing of the study during the COVID-19 pandemic, we faced difficulties in getting stakeholders to participate.

### 4.3. Comparison with existing literature

To date, there are limited qualitative studies that explore stakeholders’ views and experiences on implementation of POCTs for CA-ARTI. Qualitative studies conducted in the UK involved stakeholders with similar roles to our study but took place prior to the pandemic (7, 13) while another focussed on pediatric ambulatory care (14). These studies, however, reported similar findings to our UK data and extended to our EU and Belgian data (7, 13). Policy-level facilitators included robust reimbursement policies and incentivizing the use of POCTs in primary care (23, 24). In line with our study, one study in the UK posited that a viable strategy to facilitate uptake of POCTs would be to procure them on a national-level and supply primary care practices with them (7). On the other hand, studies also showed that stakeholders were concerned that reimbursement policies and financial incentivization may lead to the inappropriate use of POCTs as seen in our data (7, 23).

Studies from low resource settings on implementing POCTs for infectious diseases, showed that stakeholder engagement is necessary, however, this can be a difficult and time-consuming process (25, 26). In addition, studies in the UK demonstrated that stakeholders believed that there is still a lack of awareness of the existence of POCTs and that engaging with end-users is crucial (13). Across our data, stakeholders asserted that clinician champions are needed to contribute toward adoption, being the main end-user. Our data corresponds to other literature where clinicians, as early-adopters, have the potential to raise awareness, encourage other primary care practices to adopt novel POCTs, and support the generation of new evidence on POCTs (7, 13, 27). Generating new evidence is particularly important as stakeholders in our study, especially from Belgium and the UK, emphasized that national data is necessary for successful implementation (13). Moreover, our study illustrated that stakeholders in Belgium and the UK had doubts on the cost-effectiveness of POCTs in optimizing antibiotic prescribing behaviors and while acknowledging that demonstrating cost-effectiveness is challenging, they still wished to see national data (24).

Existing literature has indicated that implementing POCTs in primary care can have an impact on the way in which practices run with concerns arising on limited staff, space, and consultation times (7, 13, 24, 28, 29). In addition, our data suggested that in Belgium, special attention needs to be paid toward the role of laboratories that may feel that POCTs in primary care encroaches in their domain. While our UK participants did not iterate this, other studies in the UK and in low-income countries pointed out that laboratories may be barriers to POCT implementation as it could result in a loss of income for them (7, 13, 25, 30).

### 4.4. Implications for practice and future research

Stakeholders working at an EU-level, and in Belgium and the UK, believed that the burden of covering the costs of implementing and using POCTs should not fall on clinicians and patients. Policy-makers should therefore consider cost-neutral funding models, such as a lump-sum fees for diagnostics or robust reimbursement policies, that alleviate financial and logistical burden off end-users. In line with our study, other qualitative studies on POCTs for CA-ARTI illustrated that stakeholders were concerned with the inappropriate use of POCTs that would come as a result of financially incentivizing the use of these tests in practices (7). Therefore, non-financial incentives such as comparing quality indicators across practices that use POCTs, may help to encourage appropriate use of tests. To further ensure that clinicians use POCTs appropriately, proper guidance and/or training should be provided to clinicians on which target populations POCTs can be used for.

Diagnostic developers should consider identifying and engaging with relevant stakeholders who are closely involved in making impactful changes as early as possible, even during the product development stage. In addition, engaging with early adopters, such as clinicians to act as champions for new diagnostics, may galvanize interest among other clinicians (7, 13, 27). On the other hand, industry stakeholders also wished to see changes made on an EU-level concerning HTAs to avoid different processes and evaluations, and instead move toward a unified approach for diagnostics. In December 2021, a new EU regulation was adopted that will come into effect in January 2025 which focusses on joint clinical assessments and further collaborations between EU countries to reduce the duplication of work for national HTA bodies (31). Indeed, participants in our study noted that strengthening ties between stakeholders to encourage collaboration was one of the main lessons learnt from the COVID-19 pandemic to facilitate implementation of new diagnostics. Although the new EU HTA regulation does address this, a European trade association representing the diagnostic industry, issued a statement voicing their skepticism over the impact this new regulation will have in reducing barriers for implementing diagnostics (32). This suggests that additional advocacy work will be needed on a policy-level to amend the new HTA regulation to reduce some of these challenges that are specific to diagnostics.

Due to the impact that implementing POCTs may have on current organizations and workflows in primary care settings, diagnostic developers may need to consider specific characteristics for POCTs tailored for these settings such as, shorter time to result and automating results onto a patient’s electronic medical record to save time. In addition, the role of laboratories may need to be further defined if POCTs are to be adopted widely as they may be involved in validating results coming from tests in communities.

Lessons can be observed from the pandemic on how to effectively implement diagnostics, including strengthening existing relationships and communication channels. In addition, the pandemic broadened the role of pharmacists to include rapid antigen testing (33), thus, policy-makers may consider settings such as pharmacies, emergency rooms, and out-of-hours services as spaces for POCTs for CA-ARTI.

Future research for the implementation of POCTs in primary care settings should generate contextualized evidence to demonstrate the effectiveness of POCTs as countries have different healthcare systems and certain evidence may not be directly extrapolated. While it would be impractical to conduct repeated trials across all European countries, research studies should capture information on context to explain how POCTs are impacting (or not) primary care practice across a range of health systems and settings. Implementation studies, where POCTs are introduced in practice and use and adoption observed over time could be a beneficial, and a less costly approach, compared to trials. Such studies are similar to local service evaluations but applied more widely. In addition, further research may be needed on how to incentivize clinicians to use POCTs to ensure that they are appropriately used. Following successful implementation of POCTs in primary care settings, primary care practices may need to be monitored on their use of tests and their subsequent impact on antibiotic prescriptions.

## 5. Conclusion

Implementation of POCTs require changes on multiple-levels: at a policy-level in terms of robust reimbursement policies and efficiently evaluating diagnostics; at an organizational-level to embed POCTs in care pathways and primary care contexts; and at a clinician-level, to ensure POCTs are easy to use and are used appropriately. Industry engaging with stakeholders early on in the product development process could benefit them. Stakeholders requested national evidence on POCTs and industry may not find this feasible, but alternative research study designs could address this. Having European countries share evaluation assessments for diagnostics where necessary may overcome duplication of efforts. The COVID-19 pandemic has created opportunities for testing in new spaces such as pharmacies. In addition, there is uncertainty among stakeholders on the impact of POCTs on antibiotic prescribing and thus, further evidence may be needed to understand how practices adopt POCTs and monitoring how POCTs are used can inform future guidelines on successful implementation. Although stakeholders do not anticipate the expedited approval process for COVID-19 diagnostics to be applied to other diagnostics, they believe that valuable lessons can still be learnt from the pandemic.

## Data availability statement

The original contributions presented in the study are included in the article. Further inquiries can be directed to the corresponding author.

## Ethics statement

The studies involving human participants were reviewed and approved by University of Oxford Central University Research Ethics Committee. The participants provided their verbal informed consent to participate in this study.

## Author contributions

SA and ST-C were the principal investigators of this study, conceived the study, and participated in the analysis. EC, MEH, SA, and ST-C drew up the stakeholder mapping. MEH recruited, interviewed, analyzed, and drafted the manuscript. All authors contributed to the article and approved the submitted version.

## Funding

This work is being funded by the Innovative Medicines Initiative 2 Joint Undertaking (JU) under grant agreement No. 820755. This JU receives support from the European Union’s Horizon 2020 research and innovation programme and EFPIA and Bio-Rad laboratories, BD Switzerland Sàrl, Accelerate Diagnostics S.L. and The Wellcome Trust Limited. ST-C was supported by the National Institute for Health Research (NIHR) Health Protection Research Unit in Healthcare Associated Infections and Antimicrobial Resistance (NIHR200915) at the University of Oxford in partnership with the UK Health Security Agency (UKHSA).

## Conflict of interest

J-LT was employed by bioMérieux.

The remaining authors declare that the research was conducted in the absence of any commercial or financial relationships that could be construed as a potential conflict of interest.

## Publisher’s note

All claims expressed in this article are solely those of the authors and do not necessarily represent those of their affiliated organizations, or those of the publisher, the editors and the reviewers. Any product that may be evaluated in this article, or claim that may be made by its manufacturer, is not guaranteed or endorsed by the publisher.

## Abbreviations

CA-ARTI: Community-acquired acute respiratory tract infection
COVID-19: Coronavirus disease 2019
EU: European Union
HTA: Health technology assessment
POCT: Point-of-care test
UK: United Kingdom
VALUE-Dx: Value of diagnostics to combat antimicrobial resistance by optimizing antibiotic use
