# Supplementary material for: Stakeholders’ views and experiences on implementing new diagnostics in primary care to support management of community-acquired acute respiratory tract infections: a qualitative study
Source: Front Public Health. 2023 Jul 31;11:1216940. doi: 10.3389/fpubh.2023.1216940 (PMC10425264; doi:10.3389/fpubh.2023.1216940)
Supplement: Supplementary file 1 [file Data_Sheet_1.docx]

Supplementary Material

Stakeholders’ views and experiences on implementing new diagnostics in primary care to support management of community-acquired acute respiratory tract infections: a qualitative study

Melanie Eugenie Hoste^*^, Elien Colman, Marta Wanat, Gail Hayward, Jean-Louis Tissier, Maarten Postma, Herman Goossens, Sibyl Anthierens^†^, Sarah Tonkin-Crine^†^, on behalf of the VALUE-Dx study team

^†^ These authors share last authorship

*** Correspondence:** Corresponding Author: melanie.hoste@uantwerpen.be

# Supplementary File

**Topic Guide**

**Briefing**

1. Welcome and thanks to participant for agreeing to take part.
2. Introduce self.
3. This interview is for the IDEA study. The aims are described in the participant information sheet. Differences between professionals may arise from different perceptions of settings and organisations, from different priorities, and from different beliefs about healthcare provision. These differences are important to us and we value your unique perspective.
4. If at any time during the interview you do not wish to answer a question, that’s okay.
5. I would like to audio record our conversation. The recording will be transcribed, but your data will be pseudonymised. Your name and any names you mention, and any places you mention will be taken out, so that if someone read your interview transcript, they would not know who you are or where you work.
6. Your interview will remain confidential.
7. If, at any stage, you wish to stop the audio recording, please let me know.
8. Do you have any questions?

**Topics to be explored**

Below is a list of topics to be discussed. The topic guide will remain flexible with respect to what is of importance to participants, however, the key topic of stakeholders’ views and experiences of introducing new diagnostic tests to community care settings to help manage community acquired respiratory tract infections will remain the same.

1. Participant views on their organisation and its role in introducing/implementing diagnostics in community care settings.
2. Participant views or experience of supporting the delivery of COVID diagnostic testing through community/primary care services during the COVID pandemic.
3. Participant views or experience of existing processes, roles and strategies used within and across different organisations when introducing new diagnostics in community care settings.
4. Participant views or experience of (potential) barriers and facilitators to the adoption of new diagnostic tests in their community care settings.

**Example questions** (additional questions may be added during interviews following the topics above):

1. Can you briefly describe your position and role within your organization?

Prompts: What are your main responsibilities? How does your role link to community care and/or diagnostics?

1. Can you briefly describe the main goals and main activities of your organization?

Prompts: What type of organisation do you work in? How it is funded? Which other organisations do you have links with?

As part of the wider VALUE-Dx programme we are interested in supporting the adoption and sustained use of novel, state of the art, diagnostics in community care settings to help manage respiratory tract infections.

Community testing carried out during the COVID pandemic is a specific example of how novel diagnostic tests were rapidly introduced and adopted in community settings to provide additional diagnostic information to clinicians.

1. Can you tell us about any involvement you and your organisation had in providing COVID testing in community settings in your area?
2. What were the processes by which COVID testing was introduced and implemented in your country/area?

Prompts: What was similar to previous adoptions of new diagnostics? What was different? What went well in terms of implementation? What could be improved?

1. How can the introduction and adoption of COVID diagnostic testing inform the introduction of new diagnostic testing in primary care going forwards?
2. How do you think COVID diagnostics will be used going forwards? How best could they be used alongside other novel diagnostics?

As part of this project we are particularly interested in diagnostics that would include point-of-care tests which can be carried out at the time of patient presentation in community care with results being available to the clinician within minutes, hours or a day or two. They may be tests which use finger prick blood or nose/throat swabs. These tests may help to inform antibiotic prescribing decisions.

1. What are your views on these types of tests being introduced in the community care settings with which you are familiar?

Prompts: Have you any experience of developing/implementing such tests? How do you think such tests could influence practice?

1. What are the potential benefits to your organisation of implementing novel diagnostics in community settings to aid management of respiratory tract infections?
2. What are the potential disadvantages to your organisation of implementing such diagnostics?
3. How do you think the *introduction and adoption* of such tests in community care could best be supported?

Prompts: Do you in your role, or your organisation, have a role to support this? What are potential barriers and challenges to this process (political, financial, health care system, local manages/governance, guidelines, available resources, clinicians, patients)?

1. How do you think the *sustained use* of such tests in community care could best be supported?

Prompts: Do you in your role, or you organisation, have a role to support this? What are potential barriers and challenges to this process (political, financial, health care system, local manages/governance, available resources, clinicians, patients)?

1. How are such tests paid for when used in community care?

Prompts: Who pays for the test? If patients have to pay how does this work?

1. How do patients typically access such tests?

Prompts: are they available through general practice? Does the patient have to contact or attend any other healthcare setting (e.g. pharmacy, hospital).

1. What other organizations, departments within an organisation, or persons do you think would support the implementation of such diagnostics?

Prompts: What do you think these supporters would gain from supporting this? Would any organisations oppose this proposal?

1. Who are the key stakeholders to work with on implementing diagnostics in community settings, in your country?

Prompts: Whose role is central in the achievement of implementing diagnostics? Who should be in charge of which reforms? How can clinical guidelines support implementation of diagnostics?

1. In your experience, what approaches have worked best when trying to implement new technology or practice approach?

Prompts: Which types of policies or recommendations are most effective? What are the best ways of communicating new changes? Why are these successful? What can we learn from these examples?

1. What approaches have not worked well when implementing new technology or practice approach?

Prompts: Why have these been unsuccessful? What can we learn from these experiences?

1. What is your vision on a (future) implementation process of diagnostics for community acquired acute infections? How do we move from the current system towards implementation? Which strategies can be used?
2. Lastly, from a scale for 1 (not at all) to 10 (as ), how well do you think community care settings in your country are utilising diagnostics for the treatment of respiratory tract infections? Why did you pick this score? How would you explain this?

**Concluding questions:**

- Do you have any additional remarks?
- Is there something that you think we didn’t cover that is relevant to this issue / topic?
- Is there someone else you think we should talk to, that you can identify as a key stakeholder in this area?

Thank you for your time.
